# Treatment Strategy for Pyoderma Gangrenosum: Skin Grafting with Immunosuppressive Drugs

**DOI:** 10.3390/jcm11236924

**Published:** 2022-11-24

**Authors:** Mai Nishimura, Kento Mizutani, Naho Yokota, Hiroyuki Goto, Tomoko Akeda, Hiroshi Kitagawa, Koji Habe, Akinobu Hayashi, Keiichi Yamanaka

**Affiliations:** 1 Department of Dermatology, Mie University Graduate School of Medicine, 2-174 Edobashi, Tsu 514-8507, Japan; 2 Department of Oncologic Pathology, Mie University Graduate School of Medicine, 2-174 Edobashi, Tsu 514-8507, Japan

**Keywords:** pyoderma gangrenosum, dysregulation of inflammasome function, pathergy, negative pressure wound therapy, skin grafting, immunosuppressive drugs

## Abstract

Pyoderma gangrenosum (PG) is a relatively rare neutrophilic dermatosis presenting as a rapidly progressive and painful skin ulcer characterized by undermined borders and peripheral erythema. Immunosuppressive therapy is the first-line treatment for PG; however, large ulcers often take months or years to heal. Surgical treatments, such as negative pressure wound therapy (NPWT) and skin grafting, are still controversial due to the risk of inducing the pathergy phenomenon and eliciting PG development by traumatic factors. Herein, we report on four cases of PG treated with skin grafting, with or without NPWT, under the control of immunosuppressive drugs at our institution. All cases adapted well, but one case showed recurrence at the periphery of the grafted area five months postoperatively. The current patients were treated with the following doses of oral prednisolone (PSL): PSL 10 mg daily, PSL 5 mg daily + adalimumab 40 mg/week, PSL 12 mg + 6 mg of tacrolimus daily, and PSL 20 mg daily during skin grafting. No severe complications, including infections, were observed. Surgical treatments, such as skin grafting with or without NPWT, may accelerate wound healing, shorten the administration of analgesics and long-term immunosuppressive therapy, and reduce the risk of infection.

## 1. Introduction

Pyoderma gangrenosum (PG) is a rare neutrophilic dermatosis characterized by rapidly progressive and painful skin ulcers, undermined borders, and peripheral erythema. Epidemiological studies have shown that the average age of PG onset is in the mid-40s, with an incidence of a few cases per million person-years [1,2]. PG is often associated with various autoimmune diseases, most commonly inflammatory bowel disease and rheumatoid arthritis [1,2]. Recently, PG has been included in the category of autoinflammatory diseases, which are collectively characterized by recurrent episodes of sterile inflammation without circulating autoantibodies and autoreactive T cells [3]. Interleukin (IL)-1β and its receptor are overexpressed in patients with PG compared to healthy controls, which has been linked to the dysregulation of the inflammasome function [3]. Systemic steroids such as oral prednisolone (PSL; 0.5–2 mg/kg/day) [4] are first-line therapies, and immunosuppressive drugs such as cyclosporine (3–6 mg/kg daily) [5] may be used as adjuncts in refractory or severe cases. Immunomodulators such as colchicine, dapsone, minocycline, clofazimine, thalidomide, infliximab, cyclophosphamide, melphalan, chlorambucil, interferon, potassium iodide, and mycophenolate mofetil have also been used for the treatment of PG [6]. More recently, adalimumab, a tumor necrosis factor-alpha (TNF-α) inhibitor, has also been used to improve patient satisfaction [7,8,9,10]. However, large ulcers often require months or even years to heal. Surgical therapies such as negative pressure wound therapy (NPWT) and skin grafting are still controversial due to the possibility of inducing the pathergy phenomenon and eliciting PG development via traumatic factors [11]. Nonetheless, considering the emotional and physical burden on patients, the ulcer response after 2–4 weeks of treatment should play a role in determining whether the treatment intensity needs to be increased or maintained at a constant level [12]. This report describes four cases of PG treated with skin grafting with or without NPWT at our institution.

## 2. Case Reports

### 2.1. Case 1

**Figure 1:**
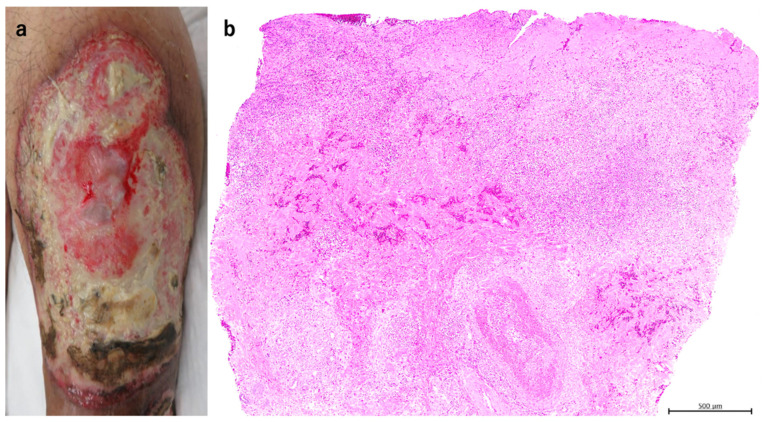

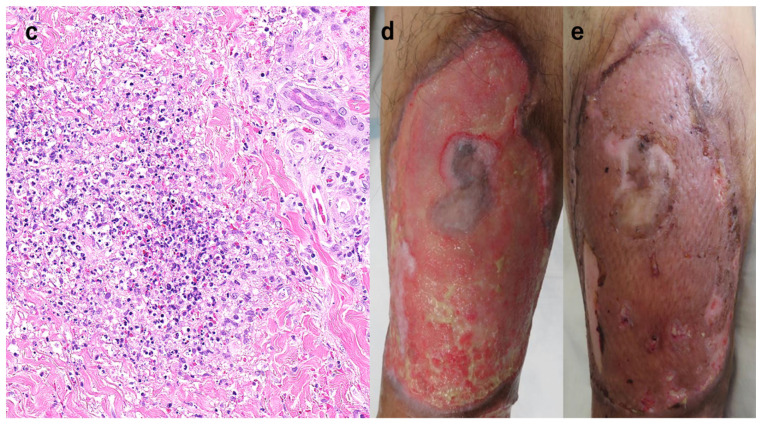
The picture of the initial examination. A 15 × 10 cm ulcer was observed on the left calf (**a**). The pathological picture of the biopsy showed neutrophil infiltration, hemorrhage, and necrosis mainly in the dermis (H & E stain ×40, (**b**), ×200, (**c**)). The picture after performing NPWT for 11 days (**d**). The ulcer shrunk and flattened, and the image after skin grafting resulted in good healing (**e**).

A male in his seventies complained of an ulcer on his left calf produced through scratching three years ago. He was treated with a topical ointment, including steroids. The ulcer would improve but then recur. The patient had a history of type 2 diabetes, myocardial infarction, and cerebral infarction, but no thrombus formation was detected in either lower leg. Previous photographs of the lesion depicted a cluster of ulcerated erythematous, purpuric vesicles mainly detected on the left calf. On admission, a large, progressive, and painful ulcer was noted (Figure 1a). Biopsy revealed neutrophilic infiltration, hemorrhage, and necrosis, seen mainly in the dermis (Figure 1b,c). Based on the clinical and pathological findings, PG was diagnosed. Treatment was started with PSL 30 mg/day (0.5 mg/kg), and the C-reactive protein (CRP) level improved from 6.1 mg/dL to 0.29 mg/dL in one week. PSL dose was reduced by 5 mg every two weeks and then by 2.5 mg after reaching PSL 20 mg daily. On day 26 after the start of PSL, NPWT (VAC ULTA^®^ therapy system; 3M/KCI, Tokyo, Japan) was started under PSL 17.5 mg/day and continued for a total of 11 days. Negative pressure was applied at −75 mmHg due to pain. The ulcer showed granulation with no apparent activity (Figure 1d). Skin grafting was performed, and 10 mg/day of PSL was administered, resulting in complete recovery (Figure 1e). No postoperative recurrences were observed.

### 2.2. Case 2

**Figure 2:**
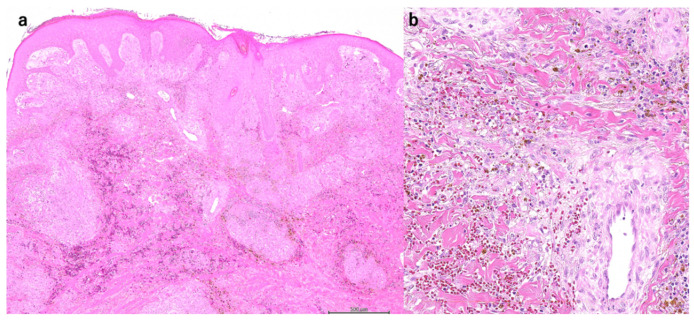

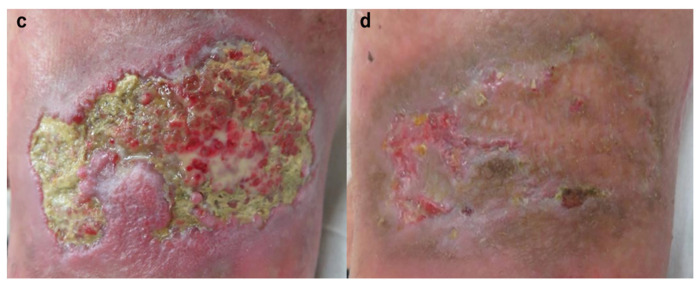
Skin biopsy showed increased vascularization and inflammatory cell infiltration, mainly neutrophils, and lymphocytes, in the dermal to subcutaneous tissue (H & E stain ×40, (**a**), ×200, (**b**)). The picture taken at the time of the re-examination showed the enlarging ulcer with yellow necrosis (**c**). The picture after skin grafting. Implantation of the skin graft was successful (**d**).

A male in his thirties had perforating and maggot-infested ulcers on both lower legs 14 months before presentation. No varicose veins or deep vein thromboses were noted in the lower extremities, and biopsy showed increased vascularization and inflammatory cell infiltration, with notable neutrophilic and lymphocytic dominance in the dermal layer extending to the subcutaneous tissue (Figure 2a,b). Based on clinical and pathological findings, the patient was diagnosed with PG. The patient was treated with PSL 10 mg/day (0.1 mg/kg) for three months, but the ulcer failed to improve. The patient has switched to adalimumab 160 mg, 80 mg after two weeks, 40 mg after four weeks, and 40 mg/week with PSL 5 mg/day resulting in ulcer reduction, but he discontinued treatment due to personal reasons. Three months later, the patient consulted again due to ulcer growth with concomitant yellow necrosis (Figure 2c). Adalimumab 40 mg/week and PSL 5mg/day were restarted. When the disease activity was sufficiently stable at CRP 0.2 mg/dL, skin grafting was subsequently performed. Implantation of the skin graft was successful (Figure 2d).

### 2.3. Case 3

**Figure 3:**
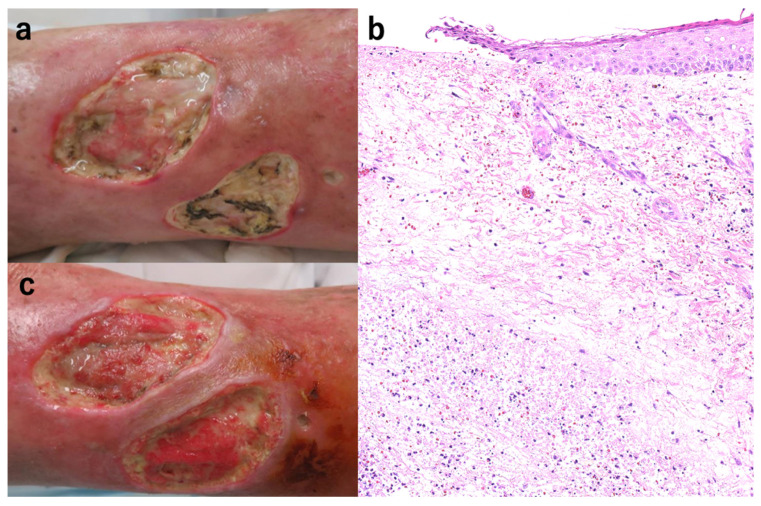

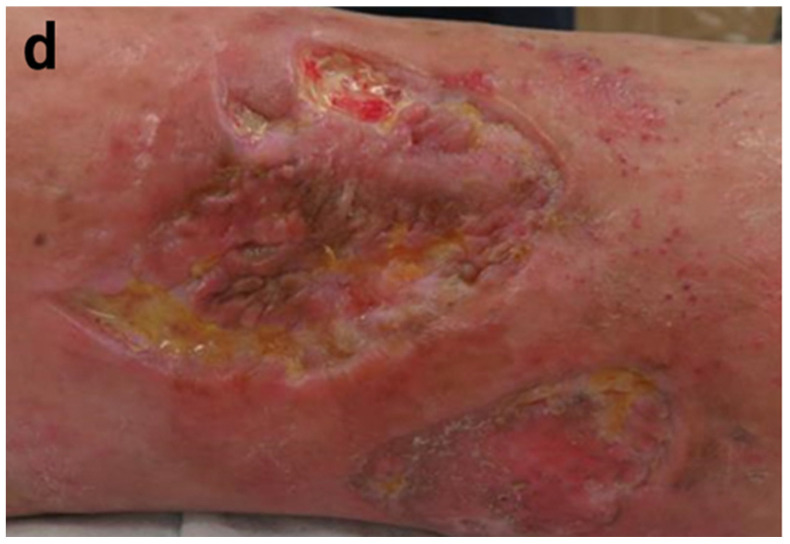
The ulcer on the right leg increased rapidly (**a**). The skin biopsy showed epidermal erosions, hemorrhage, and numerous inflammatory cell infiltrates, including neutrophils in the dermis (H & E stain ×100, (**b**)). The picture after introducing NPWT for one week (**c**) and skin grafting (**d**).

A female in her sixties complained of an ulcer on her right lower leg that appeared six months before without any precipitating factors. She was taking PSL 12 mg/day (0.26 mg/kg) and tacrolimus hydrate (TAC) 6 mg/day because of multiple sclerosis, neuromyelitis optica, and myasthenia gravis. The ulcer on the right leg had rapidly enlarged (Figure 3a). Skin biopsy showed epidermal erosions, hemorrhage, and numerous inflammatory cell infiltrates, including neutrophils, in the dermis (Figure 3b). Under the diagnosis of PG, PSL was increased to 22 mg/day (0.5 mg/kg) with TAC continuance. After one month, ulcer progression was inhibited, and PSL was reduced to 17 mg/day. After another week, the dose was reduced to 12 mg/day. NPWT (VAC ULTA^®^ therapy system; 3M/KCI, Tokyo, Japan) was started for one week under PSL (12 mg/day) and TAC (6 mg/day) (Figure 3c). Skin grafting was then performed, and the grafts were fixed with NPWT, as previously reported [13]. On follow-up, the ulcer was noted to be relatively well-controlled without relapse (Figure 3d).

### 2.4. Case 4

**Figure 4:**
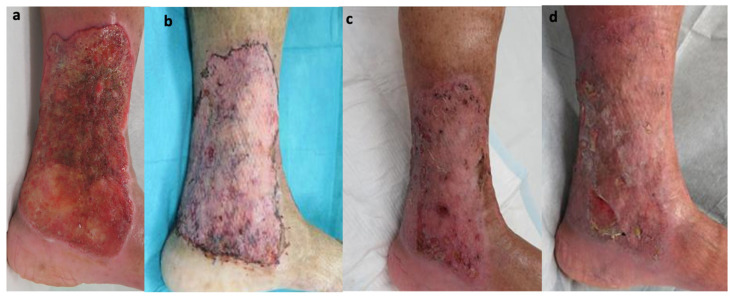
The picture of the initial examination. A 15 × 10 cm ulcer was observed on the inner foot of the left lower leg (**a**). After skin grafting, with good skin engraftment (**b**). After five months of the operation, a small ulcer re-occurred around the peripheral of grafted area (**c**), and the ulcer was unchanged at the time of 7 months (**d**).

A female in her seventies developed ulceration on her left lower leg three years ago. She had rheumatoid arthritis for 40 years and was orally treated with methotrexate 4 mg/week. A skin biopsy was performed on suspicion of rheumatoid vasculitis; however, no traces of vasculitis was found, and the ulcer rapidly enlarged following the biopsy (Figure 4a). Histology showed denaturation of collagen fibers and infiltration of inflammatory cells containing neutrophils into the dermis. Based on the clinical course and histology, the patient was diagnosed with PG. Skin grafting was performed with PSL administration at 20 mg/day (0.4 mg/kg). The skin engraftment appeared to be well-adapted (Figure 4b). However, five months after the operation, a small ulcer recurred around the periphery of the grafted area (Figure 4c). Although a weekly 40 mg dose of adalimumab was immediately started, the ulcer persisted for seven months (Figure 4d).

## 3. Discussion

A phenomenon often associated with PG, which frequently worsens after minor trauma or surgery, is called pathergy. This is due to the release of alarmin and the overproduction of inflammatory cytokines such as IL-1β, IL-6, and TNF-α when epidermal keratinocytes are injured [14]. Furthermore, pathergy is induced by the activation of dendritic cells. The production of chemokines accelerates the migration of neutrophils and the activation of T cells with an immunological shift to type 1 cytokine predominance [14]. Because healing takes more than a few years in cases of large ulcers, patients are inflicted with an emotional and physical burden, including the risk of infection, persistent pain, and wound care [15,16]. In addition, open wounds are prone to secondary infection, which may trigger further deterioration [15]. Surgical treatment may be necessary to shorten the healing time, but PG risks exacerbation due to surgical invasion. In a report of 161 cases of NPWT and/or skin grafting performed for PG, treatment was successful in 139 patients (86.3%) [15]. There were 18 (11.2%) treatment failures, of which 12 were conducted without immunosuppression. The remaining four cases (2.5%) were treated only with NPWT, which resulted in pain reduction and increased good granulation tissue, but no healing or recurrence was reported. In conclusion, NPWT and skin grafting in combination with immunosuppressive agents are very effective [15].

In this current report, surgical treatments were performed with immunosuppressive drugs resulting in good healing in all four cases, although one patient (case 4) showed recurrence at the periphery five months after the operation. Compared with the conditions of the four cases (Table 1), the site of ulcer implantation was the lower leg in all cases. The time from the onset of PG to the introduction of immunosuppressive agents was thirty-four months, one month, over ten years, and two years. The time from immunosuppressive drug induction to skin grafting was two months, fifteen months, two months, and two weeks. All patients underwent skin grafting with PSL 10 mg, PSL 5 mg + adalimumab, PSL 12 mg + TAC 6 mg, and PSL 20 mg daily. None of the cases were complicated by severe infections. CRP for all four cases at the time of surgery was 0.48 mg/dL, 0.24 mg/dL, 0.44 mg/dL, and 0.02 mg/dL, respectively. All cases were regarded with adequate inflammation control. The time from onset to surgery in cases 1 to 4 was 3 years, 15 months, >10 years, and 3 years, respectively. Cases 1 and 2 exhibited no comorbidities or autoimmune disease, whereas cases 3 and 4 had comorbidities. Unfortunately, only case 4 relapsed despite successful surgery. We considered that the reason for the recurrence of case 4 was the high dose of PSL at the time of surgery since the surgery was performed two weeks after the initiation of PSL. If high-dose steroids are administered, the maintenance dose is unknown and may recur when the dose is reduced. In fact, it has been reported that of two patients who underwent skin grafting under high-dose PSL administration, the patient whose steroid dose was reduced two weeks after surgery relapsed, while the patient whose steroid dose was maintained for six months after surgery did not relapse [17]. Because of the side effects associated with long-term administration of high doses of steroids, it may be better to reduce to the lowest steroid dose and confirm that PG does not enlarge before surgery. Additionally, case 4 was associated with congestive dermatitis, and the ulcer site extended into the joint. This was also considered to increase the risk of recurrence.

We strongly suspect that wound preparation with NPWT and skin grafting under immunosuppressive therapy is an invaluable treatment strategy for PG. Since there is no consensus on the type and dosage of immunosuppressive agents to be used in combination with skin grafting or NPWT for PG, the further accumulation of cases is essential.

## Figures and Tables

**Table 1:** Comparison of our four cases.

	Case 1	Case 2	Case 3	Case 4
Disease duration until initiation of immunosuppressive treatment	34 months	1 month	Over 10 years *	2 years *
Time from start immunosuppressive treatment to surgery	2 months	13 months	2 months *	2 weeks *
Immunosuppressant dose during surgery	Prednisolone 10 mg	Prednisolone 5 mg + adalimumab	Prednisolone 12 mg + Tacrolimus 6 mg	Prednisolone 20 mg
CRP at the time of surgery	0.48 mg/dL	0.24 mg/dL	0.44 mg/dL	0.02 mg/dL
Time from onset to surgery	3 years	14 months	Over 10 years	2 years
Other autoimmune diseases	None	None	Existence	Existence
Transplantation site	Ventral lower leg	Lateral lower leg	Medial lower leg	Medial to dorsal lower leg

* As for case 3 and case 4, it is when immunosuppressive drugs were started for PG since the patients were initially taking immunosuppressive drugs for other autoimmune diseases.

## Data Availability

The patient’s data are not publicly available on legal or ethical grounds. Further inquiries can be directed to the corresponding author.
